# Evaluation of radiodensity and dimensional stability of polymeric materials used for oral stents during external beam radiotherapy of head and neck carcinomas

**DOI:** 10.1016/j.ctro.2022.06.006

**Published:** 2022-06-18

**Authors:** Katarina Jonovic, Mutlu Özcan, Nadin Al-Haj Husain, Kiren Jan Mätzener, Ilja Frank Ciernik

**Affiliations:** a University of Zürich, Division of Dental Materials, Center for Dental and Oral Medicine, Zürich, Switzerland; b University of Zurich, School of Medicine and Dentistry, Medical Faculty, Zurich, Switzerland; c University of Bern, School of Dental Medicine, Department of Reconstructive Dentistry and Gerodontology, Bern, Switzerland; d Radiation Oncology, Dessau City Hospital, Brandenburg Medical School Theodor Fontane, Dessau, Germany

**Keywords:** Cancer, Dental materials, Dimensional stability, Radiodensity, Radiotherapy

## Abstract

**Purpose:**

Intraoral stents protect the healthy tissues from ionizing radiation during external beam radiotherapy reducing mucositis, hyposalivation and osteoradionecrosis. This study investigated the radiodensity and dimensional stability of polymeric materials for suitability in construction of intraoral stents and aimed to provide clinical guidelines.

**Methods:**

Specimens were fabricated using 4 material types namely, resin composite (ProTemp-PRO), polymethylmethacrylate (PMMA) (Enamel Temp Plus-ETP, Palapress-PAL, TAB 2000-TAB), polycaprolactone (Orfit-ORF) and silicone (Adisil-ADI, Lab Putty-LAB, Memosil2-MEM, Optosil-OPT, President Plus-PRE, Siolaplast A-SIA). They were randomly assigned to measure their radiodensity in Hounsfield Units (HU) (12x12x11mm^3^) (N_radiodensity_ = 66; n = 6) using a computer tomograph (CBCT, Toshiba Aquillon LB scanner) at baseline and after 6 weeks. The scanning protocol was applied with and without single energy metal artifact reduction (SEMAR) scans using a slice thickness of 1 and 5 mm. The same materials have been tested for their dimensional stability (µm^3^) at baseline, 1, 6, 12, 24 h, 3 and 6 weeks (14 × 4 × 2 mm^3^) (N_dimension_ = 55; n = 5 per material) using stereolithography (STL) files generated by a lab scanner (L2i, Imetric4D, Courgenay, Switzerland) and analyzed using a matching software (Geomagic ControlX 2020, 3D Systems). Data were analyzed using a paired *t*-test (alpha = 0.05).

**Results:**

Radiodensity values (HU) were significantly affected by the material classification (p < 0.05). Polycaprolactone (43.6) presented significantly lower HU values followed by PMMA (91.3–414.9) than those of silicone materials (292.8–874.5). In terms of dimensional stability (µm^3^), PMMA materials (Δ:1.53–2.68) and resin composite (Δ:2.89) were significantly more dimensionally stable compared to those of silicone materials (Δ:13.64–6.63) and polycaprolactone (Δ:-0.76) and (p < 0.05).

**Conclusion:**

For fabricating intraoral stents, when reduced radiodensity values are required polycaprolactone could be recommended as it fulfils the requirements for reduced radiodensity and dimensional stability. Among all silicone materials, OPT and MEM can be recommended based on the low HU and dimensional stability.

## Highlights

• Radiodensity values (HU) were significantly affected by the material classification where polycaprolactone presented significantly lower HU values followed by PMMA.
• Among silicone materials, OPT demonstrated the lowest radiodensity and together with MEM, more dimensional stability.
• Polycaprolactone, PMMA (except for TAB), the silicone materials (OPT and MEM) and resin composite tested were significantly more dimensionally stable compared to those of the other silicone materials (LAB, ADI, PRE, SIA) tested in this study.
• For fabricating intraoral stents during radiotherapy of head and neck carcinomas, when reduced radiodensity values are required polycaprolactone, but for dimensional stability except for some silicones (LAB, ADI, PRE, SIA) other siliconematerials (OPT, MEM), PMMA and resin composite tested could be recommended. The amount of shrinkage in dimensional stability requires clinical observations as regards to fitting of the intraoral stent in case of prolonged sessions of radiotherapy.

## Introduction

Tumours of the oral cavity, ear, nose and throat represents the seventh most common cancers worldwide where in 2018, new cases of 890.000 and 450.000 deaths were recorded [1]. Carcinomas of the oral cavity and pharynx include several subtypes with the most frequent histological type being the squamous cell carcinoma [2]. The predilection site for the development of oral cavity carcinoma is often the tongue, followed by the floor of the mouth, labial and buccal mucosa, gingiva, palate and alveolar mucosa [3], [4]. Males are more frequently affected by carcinomas of the oral cavity and pharynx compared to females [5]. The main risk factors for the development of squamous cell carcinoma of the oral cavity, hypopharynx, oropharynx and larynx are consumption of tobacco and alcoholic beverages, which act synergistically, as well as HPV infections [4], [6]. Other risk factors include malnutrition, poor oral hygiene, chronic irritation and betel nut chewing [4]. As curative therapy for the treatment of oral cavity carcinomas, surgery is considered as the gold standard, if the disease is resectable with acceptable postoperative sequelae avoiding mutilation. In advanced stages of the disease, adjuvant radiotherapy or chemoradiation is applied [7]. The most common type of radiotherapy is external beam radiation using a linear accelerator with photons, which can be highly modulated and allow to deliver tumoricidal doses while limiting the dose to the healthy neighbouring tissues to acceptable levels in the vast majority of settings [2].

The dose of ionizing radiation to the peritumoral tissue leads to mucositis, which is generally painful, and bears the risk of malnutrition and of changes or loss of taste after therapy [2], [8]. Other observed side effects are radiation caries, periodontal diseases, osteoradionecrosis, trismus and relapsing fungal diseases of the oral mucosa [8], [9]. Due to back- and side scatter of radiation during radiotherapy caused by dental metal alloys such as crowns or implants, an enhanced dose may be directed to the surrounding mucosal tissue that results in mucositis and is associated with reduced quality of life [10], [11].

All materials studied are indicated for manufacturing intraoral stents applied during ionizing radiation therapy in order to protect the intraoral healthy hard and soft tissues from mucositis and to avoid hyposalivation or osteoradionecrosis. The materials are used for better repositioning and spacing in case of metal or high Z materials in teeth.

**Fig 1:**
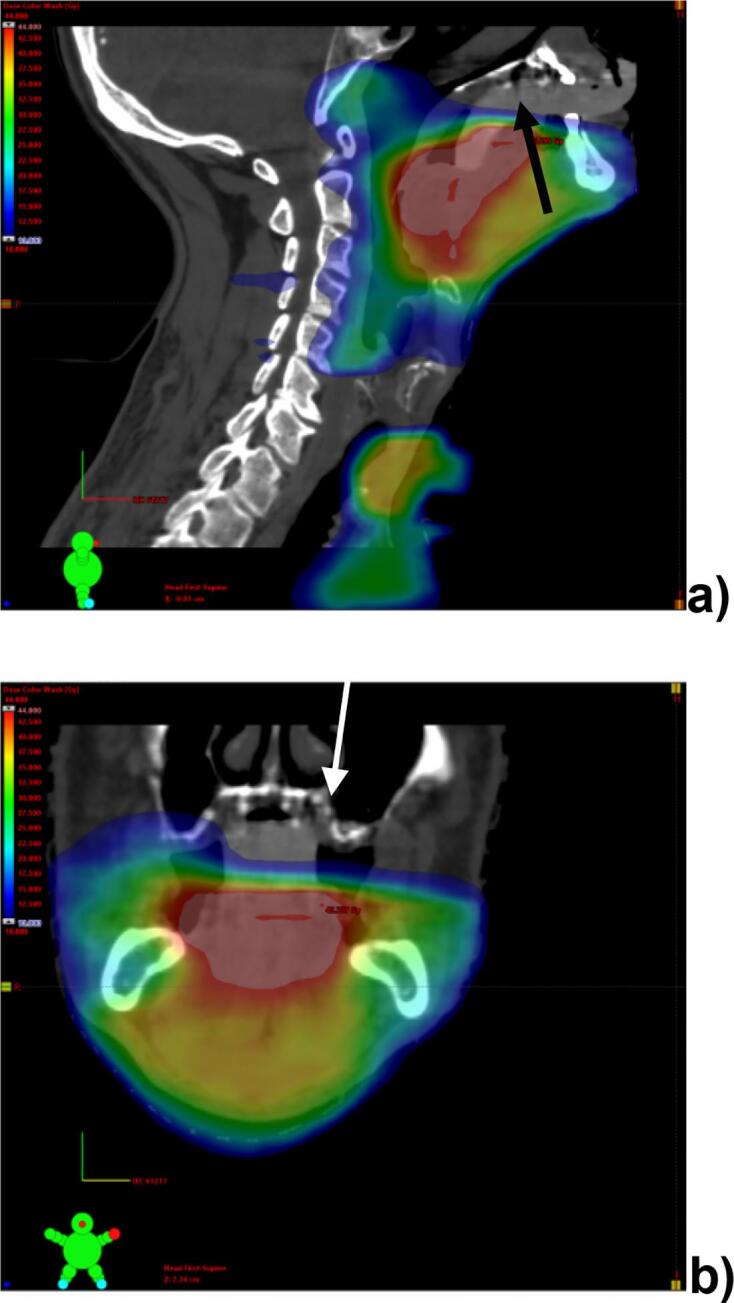
**a-b.** Planning study of a patient treated for a carcinoma of the base of the tongue with metastatic disease to the cervical lymph nodes on the right. **a)** introral stent made of silicone (Optosil; arrow black bold) to avoid dose exposure of the maxillary plate and mucosa (arrow white light). The dose distribution indicates doses exceeding 60 Gy in red, **b)** coronal view.

In order to optimize the positioning and thereby minimize the radiation to the healthy mucosa and other organs at risk (OARs) such as salivary glands, tongue and swallowing organs, intraoral stents are placed in the oral cavity during radiation. Such stents help spare the healthy tissues depending on the location of the primary tumor [12]. A representative patient case with a spacer made of silicone placed between the palate and the tongue to separate the plate from radiation while irradiating a tumour of the base-of-the-tongue is presented in Fig. 1a-b. The major goal is to avoid doses exceeding 24–30 Gy to the mucosa, minimizing the risk of xerostomia after treatment.

Typically, two categories of intraoral stents are indicated, namely, a positioning stent, which is a removable positioning aid for the tongue, the swallowing structures and the mandible/maxilla complex, ensuring reproducibility of positioning and protecting the OARs and the shielding stent, which contains shielding material and is able to absorb the beam [12]. Various pilot studies have investigated the benefit and effectiveness of intraoral stents during radiotherapy for the treatment of head and neck carcinomas [13], [14], [15], [16]. Regarding aspects of shielding, backscattering of ionizing radiation caused by metal alloys on the teeth can result in an increase in the radiation dose up to 170%, while a minimum distance of only 3 mm can effectively reduce such an effect [17]. While Obinata et al. showed that increasing the distance to the mandible during radiotherapy of carcinomas of the lateral tongue, reduces the risk of osteoradionecrosis [18], Qin et al. and Goel et al. also presented that the use of spacers may reduce severe mucositis in patients undergoing radiotherapy for head and neck cancer [19], [20]. Furthermore, intraoral stents may reduce adjustment errors in Intensity-modulated radiation therapy (IMRT) for head and neck tumours [21].

Materials used for positioning during radiotherapy should present good quality against radiation beams whereby, they should not create additional backscatter or overdosing resulting in unnecessary beam attenuation and not interfere with the planning accuracy or dose delivery. An ideal material should have a radiodensity close to 0 Hounsfield units (HU) and remain stable over time. HU values close to zero are known to cause less deviation. In addition, due to the lack of high Z materials, the HU values can be used as predictors of deviation in dose calculation. Yet, the translation into the correct physical density is essential. In order to ensure reproducible radiation treatment results, the material is also required to be dimensionally stable for the duration of the radiotherapy. Commonly, such intraoral stents are fabricated using polymeric or silicone-based materials.

To the best of our knowledge, comparative assessment of available and suitable materials for intraoral stents with a focus on both radiodensity and dimensional stability is lacking. Therefore, the objectives of this study were to (1) assess the radiodensity of the most suitable intraoral stent materials for radiotherapy planning and their stability over time and (2) evaluate the dimensional stability of the selected materials over a duration of a conventional postoperative radiotherapy of six weeks. The null hypothesis tested were that neither the material type nor the duration would affect the radiodensity and dimensional stability up to 6 weeks.

## Materials and methods

### Specimen preparation

**Fig. 2:**
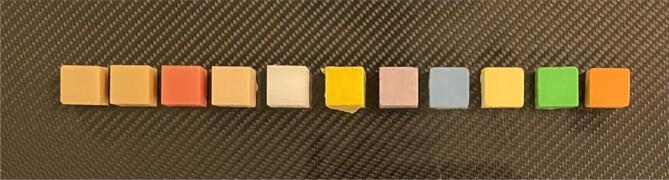
Arrangement of the tested materials according to Table 1, showing the specimens prepared for the HU measurements.

Specimens (12x12x11mm^3^) (N = 66; n = 6 per material) were fabricated using 4 material types namely, resin composite (ProTemp-PRO), polymethylmethacrylate (PMMA) (Enamel Temp Plus-ETP, Palapress-PAL, TAB 2000-TAB), polycaprolactone (Orfit-ORF) and silicone (Adisil-ADI, Lab Putty-LAB, Memosil2-MEM, Optosil-OPT, President Plus-PRE, Siolaplast A-SIA) with the aid of a metal mould according to each manufacturer s instructions (Fig. 2). In order to achieve the desired size, an individualised mould was made using duplicating silicon (Ecosil+, Dentaurum, Ispringen, Germany). The material was inverted into the mould and excess material was removed with a spatula. The PMMA materials were additionally polymerized for 10 min in a pressure pot at 45 °C to reduce the residual monomer content. The specimens were then randomly assigned to measure their radiodensity and dimensional stability. Physical and chemical characteristics, abbreviations and manufacturers of the investigated materials provided by the manufacturers are summarized in Table 1.

**Table 1:** Brands, types, chemical compositions, manufacturers and batch numbers of the materials tested in this study.

Brand	Material Type	Abbreviation	Chemical Composition	Manufacturer	Batch number
Pro Temp	Resin composite	PRO	2,2′-[(1-Methylethyliden)bis(4,1-phenylenoxy)]bis ethyldiacetat (70–80%) 1-Benzyl-5-phenylbarbitursäure (<10%) Kieselsäure (5–15%)(1-methylethylidene)bis(4,1-phenyleneoxy-2,1-ethane diyl)(1-phenylenoxy-2,2′ethoxyethanediyl)bisacetate (1–10%)t-Butylperoxy-3,5,5-trimethylhexanoat (<0.4%)	3 M Deutschland GmbH ESPE Platz D-82229 Seefeld Germany	19224–29-4 72846–00-5 68909–20-6 13122–18-4
Enamel Temp plus	Polymethylmethacrylate	ETP	Liquid: methacrylic acid ester 1,4-Butandioldimethacrylat2-(2H-Benzotriazol-2-yl) -4,6-di-tert.pentylphenolPowder: Methy-methcrylat (50–75%)1,4-Butandioldimethacrylat (2.5–10%) 2-(2H-Benzotriazol-2-yl)-4,6-di-tert.pentylphenol (<2.5%)	Schütz Dental GmbH, Dieselstrasse 5–6 D-61191 Rosbach v.d.H. Germany	80–62-6 2082–81-7 25973–55-1 80–62-6 2082–81-7 25973–55-1
Palapress Rosa	Polymethylmethacrylate	PAL	Liquid: Mehtyl methacrylate (>95%) Tetramethylene dimethacrylate (0–5%)Powder: Polymethylmethacrylate (>95%)Dibenzoylperoxide (<1%)	Heraeus Kulzer GmbH Leipziger Strasse 2 63,450 Hanau Germany	80–62-6 94–36-0 80–62-6 2082–81-7
TAB 2000	Polymethylmethacrylate	TAB	Methyl-methacrylate, Methyl 2-methylprop-2-enoat, MMA, (>90%) Ethylendimethacrylat (<10%)Methanol (<10%)N,N-dimethyl-p-toluidin (<1%)1,4-Dihydroxybenzol, Hydrochinon, Chinol (<0.1%)	Kerr Italia S.r.l. Via Passanti, 332 84,018 Scafati (SA) Italy	80–62-6 97–90-5 67–56-1 99–97-8 123–31-9
Orfit	Polycaprolactone	ORF	2-Oxepanone, Hexan-6-olide	Thermoplastic splinting Orfit Industries NV Vosveld 9a B-2110 Wijnegem Belgium	502–44-3
Adisil	Silicone	ADI	Mixture of organsiloxanes Decamethylcyclopentasiloxan (0.1–1%) Dodecamethylcyclohexasiloxane 0.1–1%)	SILADENT Dr. Böhme & Schöps GmbH Im Klei 26 D − 38,644 Goslar	541–02-6 540–97-6
Lab Putty	Silicone	LAB	Polysiloxane Alkylsilikate (15–20%)Dioctylzinndineodecanoat (5–10%)	COLTENE/Whaledent AG Feldwiesenstrasse 20 CH-9450 Altstätten	68299–15-0
Memosil 2	Silicone	MEM	Vinylpolysiloxane Octamethylcyclotetrasiloxan (0–3%)	Heraeus Kulzer GmbH Leipziger Straße 2 63,450 Hanau Germany	556–67-2
Optosil	Silicone	OPT	Polysiloxane Silanized quartz flour (25–50%)	Heraeus Kulzer GmbH Leipziger Straße 2 63,450 Hanau Germany	14808–60-7
President Plus	Silicone	PRE	Polyvinylsiloxane	COLTENE/Whaledent AG Feldwiesenstrasse 20 CH-9450 Altstätten	
Sialoplast A	Silicone	SIA	Polyvinylsiloxane Cristobalite (25–50%)Aluminium hydroxide (10–25%)	M&W Dental Müller & Weygandt GmbH Reichardsweide 40 63,654 Büdingen Germany	14464–46-1

### Radiodensity measurements

Radiodensity measurements of the specimens in Hounsfield units (HU) were accomplished using a computer tomograph (Toshiba Aquillon LB scanner, Canon Medical Syst., Neuss, Germany) at baseline and after six weeks. Specimens in each material group were scanned (Toshiba Aquillon LB scanner) using a scanning protocol with and without single energy metal artifact reduction (SEMAR) on scans with slice thicknesses of 1 and 5 mm at 120 kV.

### Dimensional stability measurements

Additional specimens were fabricated (N = 55; n = 5) (14x4x2mm^3^) from the selected materials according to each manufacturer’s specifications using a metal mould where the top layer was pressed with a glass plate to obtain a smooth surface. The homogenously mixed material was poured into the designated space of the template. The excess material was again removed with a small spatula. In case of the PMMA materials, the metal mould was coated with a thin layer of insulator (Vaseline, Verfora, 1752 Villars-sur-Glâne, Switzerland) in order to avoid a chemical bonding with the metal and at the same time to allow easy release of the specimens. Again, the PMMA materials were additionally polymerized for 10 min in the pressure pot at 45 °C. A needle was inserted into each specimen before all the specimens of each material were placed on a prepared scan body.

Dimensional stability was evaluated at baseline, 1, 6, 12, 24 h, 3 and 6 weeks. The surface of the specimens was coated with a spray (Okklusionsspray, RR Dental AG, Winterthur, Switzerland). A lab scanner (L2i, Imetric4D, Courgenay, Switzerland) was used to generate stereolithography (STL) files of scans of all specimens of each material per scan so that time differences that would have been caused by scanning the specimens separately could be avoided. The scans were made with the settings used for scanning crowns. The baseline scan was repeated after 1, 6, 12, 24 h, 3 and 6 weeks. The scans were then sliced into separate specimens using the corresponding software (Blender software, Blender Institute B.V. Amsterdam, The Netherlands). In order to eliminate the inaccuracy at the insertion point, each scan from the specimens were superimposed again using the iterative closest point algorithm [22] in Blender software, and afterwards the superimposed images were sliced using the corresponding software (Autodesk Meshmixer 3.5, San Rafael, CA, USA). This way, a flat area was generated and the inaccurately scanned area surrounding the insertion point of the needle could be eliminated. Utilizing the matching software (Geomagic ControlX 2020, 3D systems, SC, USA) the volume change in each specimen was calculated at each measured time point.

### Statistical analysis

For the statistical analyses and plots, the statistical software R version 4.0.5 (R Core Team, Vienna, Austria, 2021) including the package Tidyverse [23] and lme4 [24] were used. Data of both radiodensity and dimensional stability were analyzed using descriptive analysis and paired t-tests. Primarily the data of the measurement of the radiodensity (HU) was explored graphically and descriptively. Subsequently, observations with thickness = 1 mm and SEMAR = “with” was selected. Since the study has a repeated-measures design, paired t-tests were performed on each material to investigate whether statistically significant differences in radiodensity at baseline and six weeks (follow-up) can be found. Likewise, the data of dimensional stability measurements were primarily explored graphically and descriptively. A reduced data frame featuring only the observations at time 0 (baseline) and 6 weeks (endpoint) was generated. Because of the repeated-measures design, again paired t-tests were performed to investigate whether statistically significant differences could be found in volume at baseline and six weeks per material. The tests were performed at a 5% significance level.

## Results

### Analysis of the radiation density

**Fig. 3:**
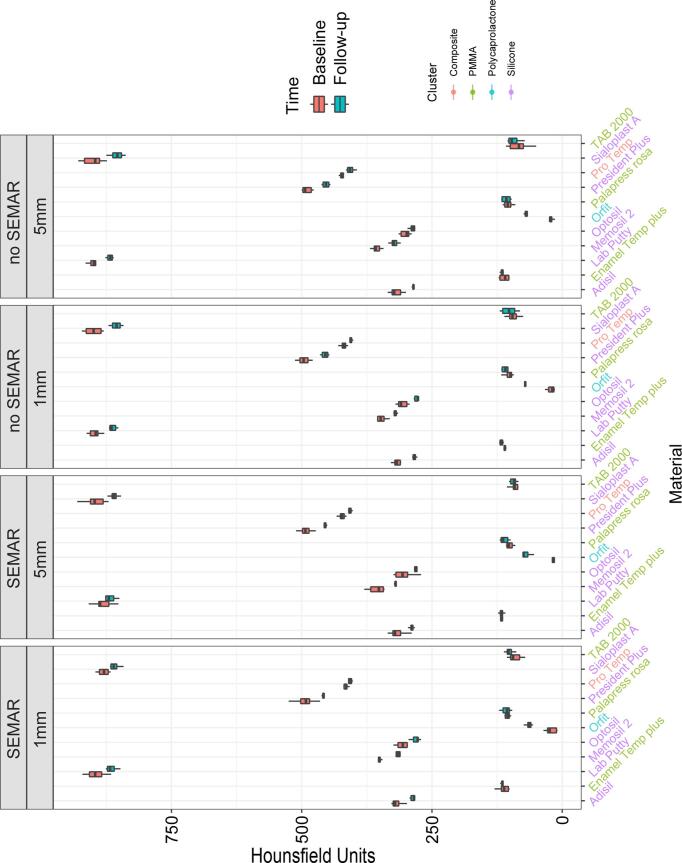
Graphical representation of the radiodensity measurements (Hounsfield-HU) as a function of material type, measuring points for the settings SEMAR = “with” and SEMAR = “no” for 1 mm versus 5 mm (Y-axis, left).

The radiodensity of the materials can be classified into 3 categories: low (up to 250 HU), medium (250–750 HU) and higher than 750 HU. Radiodensity values (HU) were significantly affected by the material classification (p < 0.05). Polycaprolactone (43.6) presented significantly lower HU values (p < 0.005) followed by PMMA (91.3–414.9) than those of silicone materials (292.8–874.5) (p < 0.05). The PMMA and polycaprolactone presented low HU values while resin composite material and 4 out of 6 silicones showed a medium radiodensity. Two silicones (SIA and LAB) on the other hand presented very high HU values. PMMA and resin composite materials seem to be more stable over time. Fig. 3 illustrates the measurement of the radiodensity in Hounsfield units (HU) of all investigated materials and Table 2 shows the evaluations of the change regarding the radiodensity in HU of the reduced data set at 1 mm, SEMAR = “with” between. According to this measurement, there has been no statistically signifant change in radiodensity for PMMA and resin composite materials over the studied duration of six weeks. Imaging parameters were not affected by the measurement of the materials. Measurements at a slice thickness of 1 or 5 mm, with or without SEMAR resulted in comparable results (Table 3).

**Table 2:** Overview of interferential statistics using the reduced data frame of the gold standard (1 mm and SEAMAR = “with”, showing the median, IOR, minimum and maximum Hounsfield (HU) values for each material type and the p-values. *p-value < 0.05 was considered significant (paired *t*-test analysis). For abbreviations see Table 1.

Material type	Brand	HU (mean)	SD	Median	IQR	min	max	p-value*
ResIn composite	PRO	414.9	9.57	412.5	13	402	433	0.2
Polymethylmethacrylate	ETP	115.9	5.73	116	5	103	124	0.8
Polymethylmethacrylate	PAL	105.8	9.3	105	11.5	90	120	0.6
Polymethylmethacrylate	TAB	91.3	9.98	92.5	8	68	106	0.2
Polycaprolactone	ORF	43.6	27.66	40	54.5	14	76	0.005
Silicone	ADI	302.7	18.38	293.5	31.8	283	335	0.002
Silicone	LAB	874.5	17.19	875.5	23	850	909	0.03
Silicone	MEM	337.2	23.07	332	31	307	380	0.00003
Silicone	OPT	292.8	18.75	289.5	24	269	324	0.0002
Silicone	PRE	474.2	21.09	469	35.2	451	511	0.01
Silicone	SIA	878.5	24.82	872	35	847	931	0.02

**Table 3:** Dimensional change (µm^3^) of the materials tested on a mean basis and standard deviation (SD) between baseline and final measurement. *p-value < 0.05 was considered significant (paired *t*-test analysis). For abbreviations see Table 1.

Material type	Brand	Mean (start)	Mean (end)	Mean Δ	SD start	SD end	SD Δ	p-value	%
ResIn composite	PRO	91.47	94.36	2.89	11.502	7.269	−4.23	0.3	3.062738
Polymethylmethacrylate	ETP	92.58	94.25	1.67	8.044	5.789	−2.26	0.4	1.771883
Polymethylmethacrylate	PAL	94.53	96.06	1.53	12.079	8.52	−3.56	0.8	1.592755
Polymethylmethacrylate	TAB	87.9	90.58	2.68	6.061	5.771	−0.29	0.04	2.958711
Polycaprolactone	ORF	93.34	92.58	−0.76	3.582	4.195	0.613	0.5	−0.82091
Silicone	ADI	100.43	114.07	13.64	6.978	9.819	2.841	0.02	11.95757
Silicone	LAB	98.73	92.1	−6.63	5.337	4.001	−1.34	0.0009	−7.1987
Silicone	MEM	101.44	102.06	0.62	18.32	17.52	−0.8	0.7	0.607486
Silicone	OPT	102.71	101.92	−0.79	7.081	6.048	−1.03	0.3	−0.77512
Silicone	PRE	96.68	104.31	7.63	11.962	9.316	−2.65	0.02	7.314735
Silicone	SIA	95.54	94.46	−1.08	8.645	9.331	0.686	0.03	−1.14334

### Analysis of the dimensional stability

**Fig. 4:**
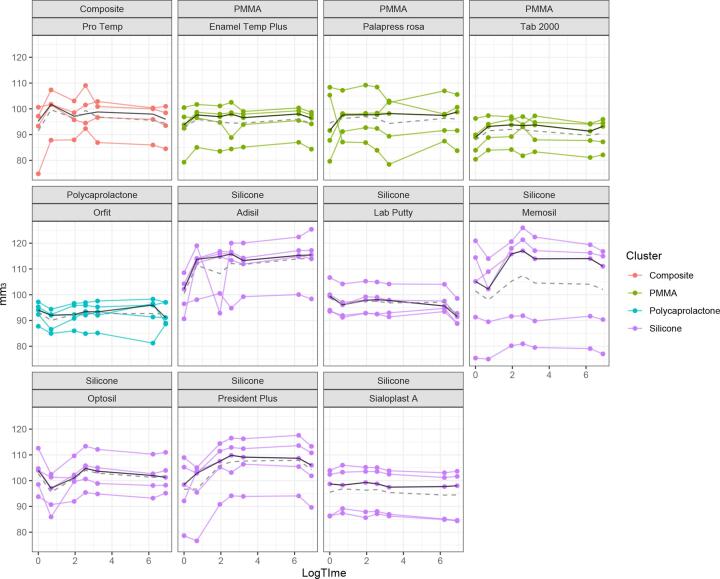
Graphical overview of the measurement of the dimensional stability as a function of time in logarithm for each material type tested. Black lines represent the mean and the dotted lines the median.

**Fig. 5:**
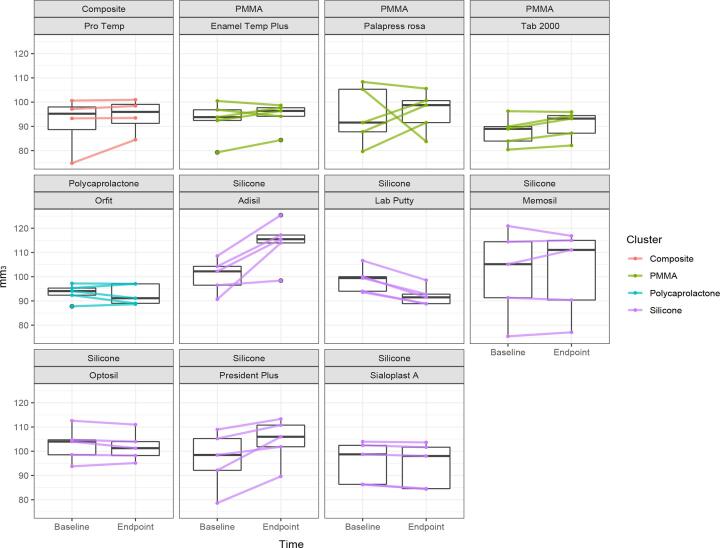
Graphical illustration of the reduced data set of the dimensional stability of the intraoral stent materials tested between baseline and the measurement 6 weeks later.

In terms of dimensional stability (µm^3^), PMMA materials (Δ: 1.53–2.68) and resin composite (Δ: 2.89) were significantly more dimensionally stable (p > 0.05) compared to those of silicone materials (Δ: 13.64–6.63) polycaprolactone (Δ: −0.76) and (p < 0.05). Fig. 4 shows the volume change in materials (n = 5) as a function of time and colour coded by material cluster. In order to better infer the short-term changes in volume, the time parameter was depicted in logarithm. In general, the materials showed the greatest fluctuation in volume up to the 4th measurement point, respectively in the first 12 h. PMMA and resin composite materials tend to increase in volume initially but thereafter remained constant over time. An exception was PAL, where larger individual fluctuations could be observed throughout the observed timespan. Silicone materials, except ADI and SIA, showed shrinkage at the beginning and expanded from the 2nd measurement time (1 h) until the 3rd measurement time (6 h). The fluctuation decreased from this timepoint onwards and the volume remained relatively stable with a slight decreasing tendency. The mean volume change and standard deviation (SD) between baseline and endpoint is presented in Table 2 (Fig. 5).

All PMMA and resin composite materials showed a slight increase in volume, which is also reflected by the rather large initial increase and then constant ratios over time in Fig. 2. The group of polycaprolactones and 3 silicone materials (LAB, OPT, SIA) showed shrinkage of the specimens over time. ADI presented the largest volume change on average with 13.64 mm^3^ and MEM the smallest with 0.62 mm^3^. The materials, TAB, LAB, PRE, SIA and ADI showed statistically significant volume changes between baseline and endpoint. Fig. 3 illustrates the volumetric change between the baseline measurement and the measurement after six weeks.

## Discussion

Intraoral stents protect the healthy tissues from ionizing radiation during external beam radiotherapy and thereby mucositis, hyposalivation and osteoradionecrosis could be reduced. This study assessed the radiodensity of the various intraoral stent materials and their stability over time and also evaluated the dimensional stability of the selected materials over a duration of a conventional postoperative radiotherapy of six weeks. Based on the results of this study, since both the material type and the duration affected the radiodensity and dimensional stability of some of the tested materials, the null hypothesis could be rejected.

The advantage of intraoral stents during radiotherapy of head and neck cancers to avoid unnecessary dose exposure to healthy tissue structures is widely accepted and reported in several case reports and case series [13], [14], [16], [19], [20], [25]. While the principles of optimal positioning are well studied, the choice of material has not been investigated systematically. During fractionated radiotherapy over weeks, evolving mucositis interferes with the use of intra-oral devices causing pain and making it difficult to maintain the initially intended position due to evolving oedema caused by the radiation.

In this study two major criteria for applicability during radiotherapy were investigated: (1) the radiodensity and (2) dimensional stability of different material categories. We further assessed the reproducibility of measurements to evaluate the stability over time, namely after six weeks corresponding to a standard period of curative radiotherapy for head and neck cancers [26]. To this point, most of the published studies used acrylic resin for the construction of stents [27], [28], [29]. Therefore, in this study three acrylic resins were included as well, but none of them have been described previously for the use as intra-orals stents.

In general, PMMA materials were characterized by low radiodensity measured in HU without relevant changes in radiodensity over a six-week period. In addition to the constant dimensional stability and the low radiodensity, especially two candidate materials for further evaluation were reserved (PAL, ETP) since both products have not been used in previous publications. The major disadvantage of PMMA materials is the logistic complexity to install the spacers. PMMA materials require a plaster model for shaping and a pressure pot for polymerization and therefore the cooperation of a dentist or a dental technician. A further disadvantage of PMMA is the rigidity of the device that easily causes additional mucosal irritation while radiation mucositis progresses as the dose increases during radiotherapy.

All studied silicone materials were characterized by higher radiodensities compared to PMMA materials, but HU values of 200 up to 300 should cause no obstacle to the dose planning process with current planning systems and algorithms. Over a treatment time of six weeks, radiation density of silicones changed more than PMMA. However, the changes were clinically not relevant and not sufficient to alter the dose distribution of the radiation plans. Currently, changes of tissue densities and anatomical changes during the weeks of radiotherapy can be corrected using adaptive replanning. Replanning includes daily Computed Tomography (CT) updates to replan and respect the current daily geometries for dose delivery. A major advantage of the silicone is the handling, as it is easy and applicable straightforward to create an individual spacer device during RT planning with a CT. Regarding handling, silicones do not need a plaster model to be moulded into the desired shape. All required steps can be performed directly by the radiation oncologist or therapists. Due to the haptic, silicones seem more compatible with progressive mucositis during RT. Two out (MEM and OPT) the 6 materials tested, appeared to be dimensionally very stable over time. With the lowest radiodensity of all silicones probed, OPT seems a suitable material in the hands of the radiation oncologists.

When measuring radiodensity, the physical density of materials is proportional to the absorption of X-rays. Denser materials appear brighter as more X-rays were absorbed (i.e. metal), and therefore the HU values are positive. Less dense materials on the other hand appear darker since less X-rays are absorbed, and therefore the HU values are negative (i.e. air) [30]. The HU value of the PMMA materials correspond approximately to the value of 103 HU described by Molteni in the case of a tube voltage of 57 keV since after calibration with the used dose, kV was supposed to be maintained stable for further planning [31]. The slightly higher value of ETP could be explained by the presence of 2-(2H-benzotriazol-2-yl)-4,6-di-*tert*-pentylphenol, which serves as an antioxidant and stabilizer against ultraviolet (UV) radiation. PMMA based materials are polymers based on acrylic acid and methacrylic acid (+ester). The powder (99%) in the volume is polymethacrylate and 90% of the liquid is methacrylate (monomer). It can be anticipated that the added pigments might have affected the HU values depending on the PMMA product. On the other hand, silicones are macromolecule chains formed by linking silicon and oxygen atoms. Eventually the difference can be explained on the grounds that the amount of filler in silicones and resin composites is higher than in PMMA materials, which is why they are less radiopaque. Polycaprolactone material (ORF) specifically developed for the treatment of surfaces or cavities in radiotherapy presented very low HU values, yet with shrinkage over time.

There are a number of other factors that can influence the HU values and therefore should be taken into consideration: the type and model of CT scanner, the stirring voltage and the type of reconstruction algorithm [32]. However, since the measurements were made with the same unit, tube voltage and measurement settings, these parameters can be neglected. Nevertheless, the values cannot be transferred to another unit as machine-related differences may have an effect upon the observed values.

Since radiotherapy of cancer patients may take longer periods of time, dimensional stability is a key feature for any materials used for therapeutic purposes in the oral cavity. Our observations are in line with the perception that polyvinylsiloxanes are classified as dimensionally very stable materials due to the absence of by-products generated during the setting reaction of addition silicones [33]. The fluctuation of the materials at the beginning can be explained by the fact that the setting reaction takes place in 2 steps. First, the material gets hard after the desired shape has been created. This reaction continues until the material is completely polymerised [34]. During this period, dimensional changes can occur, which can also be observed with practically all materials of this group.

In this study, certain silicone materials showed contraction while others expansion at the beginning. This observation is in contradiction with the observations of Aalaei et al. who reported that all silicone materials tested showed contraction in the beginning [35]. Reasons for the variations in this observation can be explained by the difficulty for the scanner to scan the lower side of the specimen where the needle was inserted. An attempt was made to correct this inaccuracy by superimposing all scans of a specimen and then removing the lower part, that is the inaccurately captured part of the scan. As an additional factor for the difference between volumes of the specimens, the cutting procedures of the scans with the software Meshmixer must be taken into account in interpreting the results. Likewise, the results could also show differences when the materials are cast on a plaster model as in the study of Aalaei et al [35]. Such a transfer in the workflow introduces errors and influences the precision of the stent material.

In the present study, a selection of commonly used dental materials in prosthetic dentistry were assessed and many non-tested materials might have resulted in a different appreciation. For example, polyethers or vinylpolyethersiloxanes have not been tested for their suitability for use as an intraoral stent. The variations in dimensional stability in the form of shrinkage or expansion could be attributed to the additives such as alkylsilicates in the polysiloxane (LAB) or cristobalite, aluminium hydroxide in polyvinylsiloxane (SIA). A more comprehensive assessment of materials could be beneficial and influence the choice of material. Furthermore, a material compatible with 3D-printing techniques (CAD/CAM technology) and off-line production from a suitable planning CT merits further investigations. Regarding materials, additional characteristics such as properties under variable conditions such as humidity or resistance to chewing stress could further help for choosing the optimal material.

An ideal material for the fabrication of an intraoral stent is characterised by a low Hounsfield Unit value, good dimensional stability, and easy handling. The PMMA materials used in this study fulfil the first two requirements best. However, material rigidity often becomes a major disadvantage with evolving mucositis over the weeks of radiotherapy. The silicones in general seem to be more suitable due to easy handling despite the higher radiodensity. Therefore, silicones merit further development where their geometric configuration could be optimized for sparing healthy oral tissues during radiotherapy.

## Conclusions

From this study, the follow could be concluded:

1) Radiodensity values (HU) were significantly affected by the material classification where polycaprolactone presented significantly lower HU values followed by PMMA.
2) Among silicone materials, OPT demonstrated the lowest radiodensity and together with MEM, highest dimensional stability.
3) Polycaprolactone, PMMA (except for TAB), the silicone materials (OPT and MEM) and resin composite tested were significantly more dimensionally stable compared to those of the other silicone materials (LAB, ADI, PRE, SIA) tested in this study.

## Clinical relevance

Among materials tested for fabricating intraoral stents during radiotherapy of head and neck carcinomas, in terms of reduced radiodensity, polycaprolactone performed best, but for dimensional stability except for some silicones (LAB, ADI, PRE, SIA) other silicone materials (OPT, MEM), PMMA and resin composite tested could be recommended. The amount of shrinkage in dimensional stability requires clinical observations as regards to fitting of the intraoral stent in case of prolonged sessions of radiotherapy.

### CRediT authorship contribution statement

**Katarina Jonovic:** Conceptualization, Methodology, Validation, Formal analysis, Investigation, Resources, Data curation, Visualization. **Mutlu Özcan:** Conceptualization, Methodology, Validation, Formal analysis, Investigation, Writing – original draft, Writing – review & editing, Visualization, Supervision, Project administration, Funding acquisition. **Nadin Al-Haj Husain:** Data curation, Writing – original draft, Visualization. **Kiren Jan Mätzener:** Data curation, Writing – original draft, Writing – review & editing, Visualization. **Ilja Frank Ciernik:** Conceptualization, Methodology, Validation, Formal analysis, Investigation, Writing – review & editing, Supervision.

## Declaration of Competing Interest

The authors declare that they have no known competing financial interests or personal relationships that could have appeared to influence the work reported in this paper.
